# Genome-Wide Association Analysis of Grain Hardness in Common Wheat

**DOI:** 10.3390/genes14030672

**Published:** 2023-03-08

**Authors:** Xianfang He, Maoang Lu, Jiajia Cao, Xu Pan, Jie Lu, Li Zhao, Haiping Zhang, Cheng Chang, Jianlai Wang, Chuanxi Ma

**Affiliations:** 1 Key Laboratory of Wheat Biology and Genetic Improvement on Southern Yellow and Huai River Valley, Ministry of Agriculture and Rural Affairs, College of Agronomy, Anhui Agricultural University, Hefei 230036, China; xianfanghe@126.com (X.H.); lumaoang@126.com (M.L.);; 2 Institute of Crop Research, Anhui Academy of Agricultural Sciences (AAAS), Hefei 230031, China

**Keywords:** common wheat, hardness index (HI), genome-wide association analysis, *PIN* gene, method of assignment

## Abstract

The grain hardness index (HI) is one of the important reference bases for wheat quality and commodity properties; therefore, it is essential and useful to identify loci associated with the HI in wheat breeding. The grain hardness index of the natural population including 150 common wheat genotypes was measured in this study. The phenotypic data diversity of HI based on four environments and the best linear unbiased prediction (BLUP) was analyzed. The results showed that the grain HI of the natural population ranged from 15.00 to 83.00, the variation range was from 5.10% to 24.44%, and the correlation coefficient was 0.872–0.980. BLUP value was used to grade and assign the grain HI to hard wheat, mixed wheat, and soft wheat, and the assigned phenotypes were used for genome-wide association analysis. Two types of grain hardness index phenotypic values were used for genome-wide association analysis (GWAS) using a 55K SNP array. A total of five significant association loci (*p* < 0.001) were excavated, among which four loci could be detected in three or more environments. They were distributed on chromosomes 1A and 7D, and the phenotypic contribution rate was 7.52% to 10.66%. A total of 48 sites related to grain hardness were detected by the assignment method, among which five were stable genetic sites, distributed on chromosomes 1A(2), 3B(1), 4B(1), and 7D(1), with phenotypic contribution rates ranging from 7.63% to 11.12%. Of the five loci detected by the assignment method, two stable loci were co-located in the phenotypic mapping results of the hardness index. One of the loci was consistent with previous reports and located on chromosome 1A, and one locus was unreported on chromosome 7D. Therefore, it may be a feasible attempt to use the assignment method to conduct genome-wide association analysis of the grain hardness index. In this study, a total of five genetic loci for grain hardness stability were excavated, and two of the loci were located in the two phenotypic values, two of which were not reported.

## 1. Introduction

Wheat (*Triticum aestivum* L.) is one of the most important cereal crops and provides approximately 20% of the dietary calories for humans worldwide, and it is the most widely cultivated food crop in the world with the largest variety of processed foods [1]. Wheat is one of the most important staple crops in China, with a planting history of more than 4500 years [2]. In recent years, with the diversification of food consumption, the market demand for high-quality special wheat has increased, and some regions have seen orders. The development of high-quality special wheat will be strongly promoted by the new modes of production, special collection and storage, and the change in market demand [3]. As one of the important indexes in this new model, grain hardness is given increasing attention.

Grain hardness is a key trait contributing to wheat processing quality and often correlates with other important traits, such as thousand-grain weight (TGW) and grain bulk density [4]. The hardness index (HI) is also an important reference for grading and pricing of international commercial wheat [5]. Therefore, exploring the genetic mechanism that underpins stable genetic loci of wheat grain hardness is very important to improve the quality and commodity properties of wheat.

The accessions with kernel HI <45 were classified as soft wheat, whereas the accessions with kernel HI >60 were classified as hard wheat. The accessions with HI between 45 and 60 were mixed wheat by the standard method (GB1351-2008) [6]. Grain hardness measurement methods include SKCS ( single-ernel characterization system), PSI (particle value number method), NIR (near infrared measurement method), HI (hardness index) [7,8], etc. Among them, SKCS is a widely used method for the determination of wheat grain hardness worldwide [9]. Soft wheat is less resistant to crushing, and the flour has a finer texture with more free starch granules, less damaged starch, and less water absorption, which avoids the formation of complete gluten when mixing and stirring the batter; therefore, soft wheat is suitable for making cakes, biscuits, and south steamed stuffed buns [5,10]. Hard wheat exhibits high resistance to crushing, the endosperm structure is relatively compact [11], the flour has coarse-textured with more damaged starch, and it can absorb more water than flour from soft wheat, making it better for food such as bread and noodles [12]. Because of the diversity of end-uses and the difference in food culture, the breeding targets of wheat grain hardness are not the same in different regions. For example, soft and waxy pasta made with soft wheat is preferred in southern China [13]. People in northern China prefer products with stronger gluten, and consequently, hard wheat is planted more widely [14].

These results demonstrated that the grain hardness of wheat is mainly affected by its own genotypes, but was less affected by environment and interaction [15]. Grain hardness is a complex trait controlled by multiple aspects, which have been found on 21 chromosomes of wheat [16,17,18,19]. The homologous genes *puroindoline a* (*Pina*) and *puroindoline b* (*Pinb*), located at the *Ha* locus on chromosome 5DS, are a pair of major contributing genes of wheat grain hardness, and their phenotypic explanation rate can be as high as 80% [20]. In addition to the *Ha* locus, many quantitative trait loci (QTLs) affecting grain hardness have been identified in different populations. Wang et al. (2012) conducted a genome-wide association study (GWAS) of recombinant inbred lines (RILs) composed of 164 materials and detected six QTLs related to grain hardness on 1BS, 4BS, 5BS, 2DS, 4DS, and 5DL, which phenotypic variation explanation ranged from 3.70% to 50.31% [18]. Li et al. (2012) detected 16 and 14 QTLs for grain hardness in the two RILs populations, respectively, with phenotypic variation explanation of 2.25–8.33% and 4.39–12.21%, among which the *QKH.Wy-22.3* gene was found to explain the highest phenotypic variation of 10.41–12.21% [21]. Maria Itria Ibba et al. mapped kernel hardness in a soft durum wheat population derived from the cross between the varieties “Creso” and “Langdon 1-678” and two major significant regions were identified on chromosomes 3AL and 6AS, which each was responsible for an additive effect of six HI units [22]. 

The QTL was based on biparental mapping that required the construction of segregating populations by crossing parental lines, which required a lot of time to obtain stable traits. As the cost of next-generation sequencing (NGS) decreases, SNPs are gradually beginning to be applied in plant breeding. GWAS based on linkage disequilibrium (LD) is a mainstream method for mining prediction genes at present. It overcomes the limitations of bi-parental QTL, shorts breeding time, reduces energy cost, and has a high resolution of mapping, allowing researchers to delineate the prediction region accurately, in order that the process of crop breeding accelerated [23]. A large number of genetic loci related to grain hardness have been found through genome-wide association analysis of natural populations by high-density SNP arrays. For example, Hu et al. (2021) analyzed 171 natural populations composed of wheat varieties by GWAS using a wheat 90 K array, and detected seven single nucleotide polymorphism (SNP) sites that are stably associated with wheat grain hardness [12]. The phenotypic contribution rate ranged from 6.67% to 11.79%. Yan et al. (2021) used a 55 K array to analyze grain hardness under five environments with a mixed linear model (MLM) and discovered 14 significant SNPs, which were detected in two or more environments [24]. *AX-110951756*, located at 7.08 Mb on chromosome 3B, had the highest explanation rate of 4.73–8.11%. In addition, GWAS also plays an important role in wheat grain morphology [25], plant height [26], and disease resistance improvement [27].

In this study, a natural population consisting of 150 wheat genotypes was used as the experimental material. The 55 K SNP array of wheat combined with the grain hardness index phenotype and classification assigned values of four environments in four years was used for GWAS, in order to mine the stable and significant genetic loci for wheat grain hardness. The experimental results provide a theoretical basis for molecular breeding of wheat hardness and necessary reference for wheat production, scientific research, purchase and sale, and processing.

## 2. Materials and Methods

### 2.1. Plant Materials

One hundred and fifty wheat accessions were collected from the Crop Research Institute of Anhui Academy of Agricultural Sciences (Table S1). In the 2017 and 2019 wheat growing seasons, the natural population was planted at the experimental station of the Crop Research Institute of Anhui Academy of Agricultural Sciences in Taihe county (33°250 N, 115°642 E), Anhui Province, China, for two consecutive years, named E1 and E2. In the 2019 and 2021 wheat growing seasons, the trial was performed at Funan Research Base experimental station of the Crop Research Institute of Anhui Academy of Agricultural Sciences in Funan county (32°761 N, 115°692 E), Anhui Province, China, for two consecutive years, named as E3 and E4, respectively. In each location, field trials (lines) were conducted in a plot 2 m long, each consisting of five rows spaced 0.25 m apart in randomized complete blocks with three replications. The field materials were sown on October 15 of each year and harvested on May 31 of the following year at each experimental station during 2017–2020. Field management followed local agricultural practices.

Statistics of average temperature, accumulated temperature, average relative humidity, monthly precipitation, monthly sunshine duration, and monthly days of rainfall of four cultivation environments are shown in Table S2. According to the sowing time of each year, the average temperature, accumulated temperature, average relative humidity, monthly precipitation, monthly sunshine duration, and monthly days of rainfall of four cultivation environments data of October are counted from the 15th to the 31st, and the data of November to May are counted in the whole month (Data from Anhui Meteorological Bureau). 

### 2.2. Grain Hardness (HI) Measurement and Grading 

From each of the 150 trials, 100 kernels were analyzed for grain hardness (HI) using the single 91 kernel characterization system (SKCS) 4100 (http://www.eectech.com.cn/productdetails.html?product_id=72 (accessed on 20 July 2018)) according to the AACCI method 55-31 (AACCI, 2010). The experiments were repeated independently three times, and kernel samples were collected at different times. The grain hardness index (HI), the higher the value, the higher the hardness [28]. The *R* (https://www.r-project.org/ (accessed on 15 August 2022)) “lme4” software package was used to calculate of best linear unbiased prediction (BLUP) [29]. The value of BLUP was used to divide wheat into hard wheat, mixed wheat, and soft wheat which, were assigned as 1, 2, and 3, respectively, according to GB/T1351-2008 (2008) [6].

### 2.3. Statistical Analysis

The Excel 2020 (https://www.microsoft.com (accessed on 20 August 2022)) was used for statistical analysis of different grade material grain hardness and calculating the minimum, maximum, average, and coefficient of variation. The *R* language “GGally” package was used to calculate correlation [30], and generalized heritability [24]. The calculation formula is defined as *H*^2^ = σg^2^/(σg^2^ + σge^2^ + σe^2^/n). Where σg^2^ is the genetic variance, σge^2^ is the variance of genotype–environment interaction, σe^2^ is the environmental variance, and n is the number of environments.

### 2.4. Chip Typing and Population Structure Analysis

The kernel DNA was extracted using the SDS method after the field material matured naturally. The DNA quality was assessed by 1.0% agarose gel electrophoresis, and the DNA concentration was measured with a NanoDrop™ND-2000 spectrophotometer [31], and 150 wheat materials were scanned using Affymetrix Axiom 55K array (Beijing Boao Jingdian Biotechnology Co., Beijing, China). Illumina’s Genome Studio Software was used for the original SNP typing of the samples. Markers with a filtration deletion rate of more than 20% and a minimum allele frequency (MAF) of less than 5%. High-quality SNP markers were retained for subsequent analysis.

### 2.5. Correlation Analysis

The mixed linear model (MLM) in *R* language “Gapit3” was used for genome-wide association analysis [4]. At the *p* < 0.001 level, it was considered that the marker was associated with the trait. The 95% *r*^2^ value was used as the threshold to estimate the LD attenuation distance, and the sites within the same LD attenuation distance were regarded as the same locus [32].

### 2.6. Candidate Gene Screening

SNPs associated with three or more environments are considered stable loci. Genes are predicted within a distance of 500 Kb of upstream and downstream around the stable site, and candidate gene functions are annotated [33] according to wheat TGAC1.0 (http://plants.ensembl.org/hmmer/index.HTML (accessed on 12 November 2022)).

## 3. Results 

### 3.1. Phenotype Analysis

We harvested 150 genotypes and analyzed them for kernel hardness. The majority of the 62 genotypes were classified as soft wheat, 51 were classified as hard wheat, and 37 were a mixture, based on the BLUP-mean value of the average value under four environments on SKCS HI distribution parameters described above (Table S1).

The HI of the natural population ranges from 15.00 to 83.00 with a variation factor of 5.53 indicating that the grain hardness of the natural population has extensive phenotypic variation under E1, E2, E3, and E4 environments (Table 1). The variation ranges of the three wheat types were 15.59–24.44%, 5.34–10.28%, and 5.10–10.55%. The minimum values are all 15.00 of soft wheat under E1, E2, and E3 environments, whereas the minimum, maximum, mean, and standard deviation of E4 were the maximum of four environments, which were 21.00, 64.00, 32.69, and 7.99. For mixed wheat, the minimum values of E2, E3, and E4 environments were less than 45.00. The maximum values of the other environments were higher than 60.00 except E4, reaching the level of hard wheat. The variation coefficient of BLUP of hard wheat was the smallest. The HI variation range of hard wheat was 38.00–83.00. The standard deviation of hard wheat under E3 was a maximum of 6.97, and the BLUP was a minimum of 3.26.

### 3.2. Variance and Correlation Analysis

**Figure 1:**
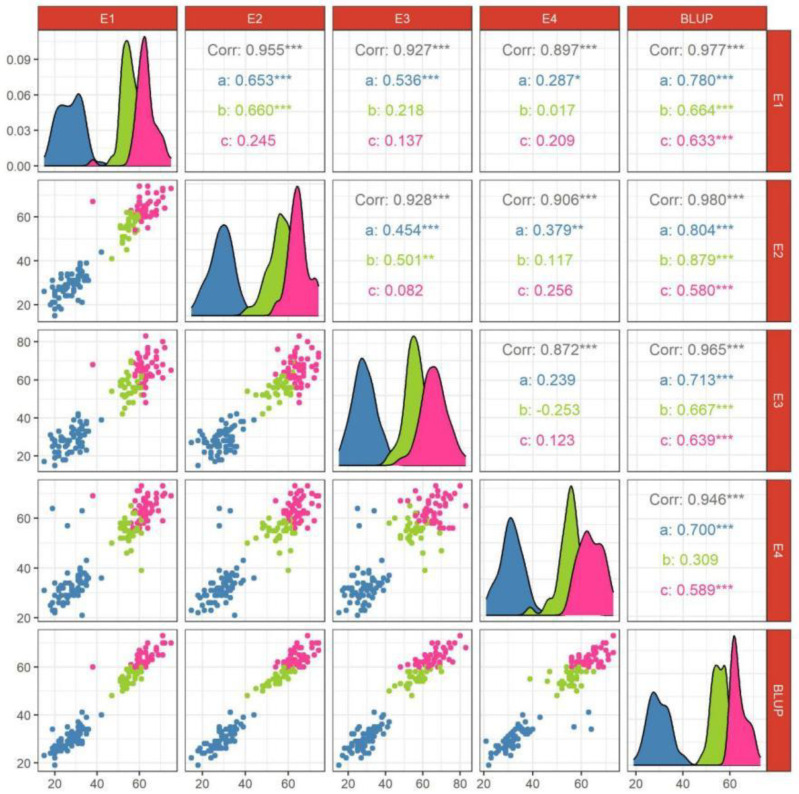
Correlation of soft wheat, mixed wheat, and hard wheat in different environments. Note: a: soft wheat; b: mixed wheat; c: hard wheat. The curves represents normally fitted distribution plots for different kinds of wheat. *: Significant at *p* < 0.05; **: Significant at *p* < 0.01; ***: Significant at *p* < 0.001.

The difference between the genotype and environment of HI of soft wheat reached *p* < 0.001 (Table 2), indicating that both genotype and environment had a very significant effect on soft wheat. HI of mixed wheat and durum wheat was significantly affected by genotype (*p* < 0.05) but not by environment or genotype × environment, which showed that HI of mixed wheat and durum wheat was mainly affected by genotype but not by environment or genotype × environment. The generalized heritability of soft wheat, mixed wheat, and hard wheat was 0.81, 0.71, and 0.63, respectively, indicating that the phenotypic variation of the three types of wheat was mainly controlled by genotype (Table 2). The grain hardness was significantly positively correlated between different environments, with a correlation range of 0.872–0.980 (Figure 1). The same type of wheat mostly showed a positive correlation extremely significant under different environments. Soft wheat showed an extremely significant positive correlation under 80% of the environment, mixed wheat did not reach a significant positive correlation level under 50% environment. There was a significant positive correlation between the BLUP values of soft wheat and hard wheat under each environment, and the correlation range was 0.239–0.804 and 0.082–0.639, respectively (Figure 1).

### 3.3. Grain Hardness Loci Identified by GWAS

**Figure 2:**
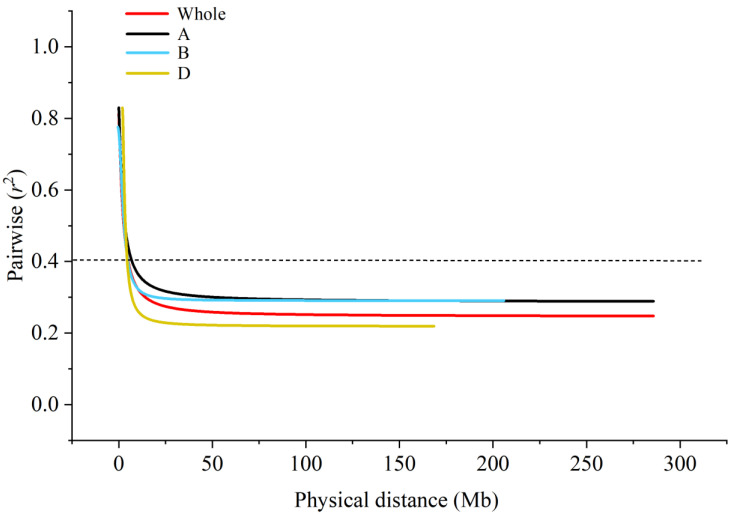
Linkage disequilibrium (LD) decay across the whole wheat genome, A, B, and D sub-genomes within the association panel consisted of 150 wheat varieties (lines) over a 300 Mb physical distance. The horizontal line indicates the critical value of *r*^2^.

**Figure 3:**
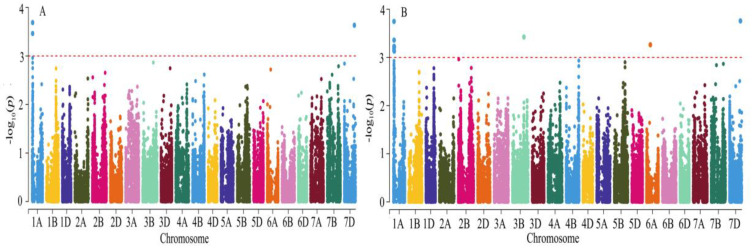
Manhattan plots for best linear unbiased predictions (BLUP) of HI of 150 wheat varieties (lines) by the mixed linear model (MLM). (**A**) Manhattan chart of hardness index; (**B**) Manhattan chart of assignment method.

Association analysis between phenotypic traits and SNP was performed using the MLM model. The *LD* attenuation distances of genomes A, B, D, and the whole genome were calculated to be 9, 4, 9, and 8 Mb, respectively (Figure 2). In this study, the LD attenuation distances of the whole genome were used as the criteria for determining effective loci. A total of two loci were found significantly correlated with grain hardness under four environments. The phenotypic explanation rate of a single locus ranged from 7.52% to 10.66%, and the average phenotypic contribution was 8.72%. The MLM model was used for GWAS of the assigned grain hardness. A total of five loci were found significantly correlated with grain hardness. The phenotypic contribution rate of a single locus ranged from 7.63% to 11.12%, the average phenotypic explanation was 8.61%. These loci were distributed on chromosomes 1A (2), 3B (1), 4B (1), and 7D (1) (Figure 3). GWAS of HI showed (Table 3) that *AX-111028882* on 1A and *AX-110005500* on 7D were detected to be significantly associated with seed hardness in all environments, with phenotypic contributions ranging from 7.63% to 10.66% and 7.52% to 10.57%, respectively. A total of five stable loci significant correlated HI were identified by genome-wide association analysis of assigned grain hardness. Marker-trait association *AX-108789085* identified by grain hardness index and the marker-trait association *AX-111028882* identified by assigned value was located within the same attenuation distance, considered as the same loci. The value of *p* ranged from 8.12 × 10^−5^ to 9.83 × 10^−4^. The *AX-110005500* on chromosome 7D had the highest phenotypic contribution rate of 11.12%, wjereas *AX-111673206* on chromosome 4B showed the lowest explanation rate of 7.63%. The 24.86–32.43 Mb position on chromosome 1A contains a large number of SNP loci significantly associated with grain hardness index. It was considered that there were major effect loci on chromosome 1A controlling grain hardness.

### 3.4. Functional Prediction of Candidate Genes

The stable genetic SNP loci significantly associated with wheat grain hardness were searched in the database, and the candidate genes were predicted and analyzed. As shown, a total of five genes possibly related to wheat grain hardness were obtained (Table 4). *TraesCS1A01G045700* encodes *Glutathione S-transferas*; *TraesCS1A01G062500* and *TraesCS7D01G448100* encode protease inhibitor, seed storage/lipid transfer protein family protein); *TraesCS3B01G386000* encodes *O-Glycosyl hydrolases* family 17 protein, *TraesCS4B01G372800* encodes the F-box family protein.

## 4. Discussion

### 4.1. Phenotypic Variation of Wheat Grain Hardness

Wheat is one of the most important crops in the world, and the quality traits of wheat have been genetically improved by breeders from different directions and angles. In this study, the BLUP values of 150 materials in four environments were used to classify the grain hardness, which could effectively correct the deviation caused by random block setting and environmental effects [35], and provide a certain reference for the subsequent assignment method. Soft wheat showed the largest range and coefficient of variation under E1, E2, E3, E4, and BLUP, which may be related to the difference in grain endosperm formation texture among different wheat varieties. According to Table 2, the generalized heritability of soft wheat, mixed wheat, and hard wheat was 0.81, 0.71, and 0.63, respectively, which were significantly or extremely significantly correlated with genotype, indicating that genotype was the main factor controlling grain hardness, which was consistent with the results of the Gazza study [15]. The heritability of soft wheat was the highest among the three types of wheat. Compared with mixed wheat and hard wheat, the grain hardness of soft wheat was less affected by the environment, and the phenotypic character was more stable. Under all environments (E1, E2, E3, and E4), the grain hardness index of 55 varieties reached the standard of soft wheat, they are Fanmai 5, Huacheng 863, Huaimai 22, Huiyan 912, Longke 1109, Quanmai 725, Wanmai 52, Zimai 19, Ningmai 21, Sulong128, Yangmai 15, Yangnuomai 1, Chuannong 16, Mianmai 37, and so on. There are 28 varieties of grain hardness index that reached the standard of hard wheat, and they are Anke 157, Annong 0711, Huaimai 40, Luyuan 502, Shannong 17, Tainong 19, Xinmai 26, Zheng 7698, Nuo 1012, Zhenmai 9, Zhongkemai 138, and so on. It shows that the trait of grain hardness index of these varieties was less affected by the environment and cultivation conditions.

The hardness index of wheat variety NO.46 in the E4 environment has a big difference from other environments, indicating that the grain hardness was easily affected by plant-condition. Yan et al. (2022) showed that populations with large variations in phenotypic traits, rich genetic background, and high generalized heritability were conducive to mining stable genetic loci for regulating related traits [36]. The hardness phenotypic data of this tested population met the conditions above, laying a foundation for subsequent genome-wide association analysis of grain hardness.

### 4.2. Genome-Wide Association Analysis of Wheat Grain Hardness

Grain hardness is not only controlled by the main effect genes (*Ha*) on chromosome 5DS, but also through minor genes on other chromosomes [19]. The 15K SNP array was used to identify nine significant marker–trait associations, including *PINs*, four of them are on chromosomes 2A, 2B, 5A, and 7A were novel loci (Wang junyou, 2022). According to the comparative results of these significant loci, in this present study five QTLs were found to be significantly associated with grain hardness by hardness index method and assignment method through genome-wide association analysis. *AX-111028882* was identified by the hardness method range of 27.39–31.94 Mb on chromosome 1A, and *AX-108789085* was found by assignment method range of 24.8–32.43 Mb on chromosome 1A, which was located within the same LD attenuation distance, and they were regarded as the same loci. In this study, major gene (*Ha*) controlling grain hardness on 5DS were not identified, which may be caused by differences in phenotypic values because of differences in natural population and cultivated environment. The results from this research are consistent with previous findings that grain hardness is mainly controlled by major gene locus, and small-effect gene locus on other chromosomes will affect it too [4]. Mergoum et al. (2013) had found a stable locus of grain hardness at 23 Mb on chromosome 1A, named *2013_Mergoum_3*, which was 4.39 Mb–8.94 Mb away from *AX-111028882* identified on chromosome 1A in this study, considered the same site of grain hardness for close distance [37]. The stable genetic locus of grain hardness was excavated at 42.55–49.23 Mb on chromosome 1A, and the locus *qHA1A.1* discovered by Hao et al. [34] at 54.5 Mb were close to each other, so they were considered as the same locus. It was further indicated that 23.00–54.50 MB on chromosome 1A played an important role on controlling grain hardness, which could be the focus of further research. This study and Hao et al. (2022) detected sites significantly associated with grain hardness traits at 656.90–661.26 Mb on chromosome 4B [27], indicating that the assignment method is reasonable at some extent. In this study, *AX-109815102* found on chromosome 3B and *AX-110005500* identified on chromosome 7D have not been reported, so they might be presumed to be a new stable genetic locus of grain hardness.

### 4.3. Candidate Gene Prediction

The stable genetic loci were predicted for candidate genes, among which *TraesCS1A01G045700* encoded glutathione S-transferas. Liu et al. conducted proteomic analysis of Chuanmai 66 and Shumai 969, which had extremely significant differences in grain hardness, and found that the glutathione metabolic pathway reached an extremely significant level of enrichment, which speculated that it had a certain effect on grain hardness [38]. At 42.55–49.23 Mb on chromosome 1A and 568.20 Mb on chromosome 7D screened for wheat grain hardness related candidate genes *TraesCS1A01G062500* and *TraesCS7D01G448100* in this study, which are responsible for encoding Protease inhibitors, seed storage, and lipid transfer protein family proteins (protease inhibitor/seed) storage/lipid transfer protein family protein). The expression of some protein in protease inhibitor is positively correlated with grain hardness [38]. The candidate gene encoding F-box family protein (*TraesCS4B01G372800*) was screened, and F-box protein participated in ubiquitin-protease pathway and played an irreplaceable role in organisms [39]. *TraesCS3B01G386000*, screened on chromosome 3B 606.68 Mb, encodes *O-Glycosyl* hydrolases family 17 protein. Glycylase can hydrolyze glycosidic bonds, and water-soluble glycoproteins in proteins and starches in wheat grains may also be hydrolyzed by them [40], thus affecting the hardness of wheat grains.

## 5. Conclusions

The phenotypes of grain hardness index of 150 wheat varieties from four environments in four years were measured and assigned into three categories. The grain hardness of natural population under four environments ranged from 15.00 to 83.00, the variation ranged from 5.10% to 24.44%, and the correlation coefficients ranged from 0.872 to 0.980. The phenotypic results of the two methods were analyzed by genome-wide association using 55K SNP microarray. Five stable genetic loci were identified, among which two by hardness value method and five by assignment method. Among them, *AX-111028882* and *AX-108899745* on chromosome 1A and *AX-111673206* on chromosome 4B are close to *2013_Mergoum_3*, *qHA1A.1* and *qHA4B.3* on the same chromosome reported by predecessors, so they are considered to be the same locus, respectively. *AX-109815102* identified at 606.68 Mb on chromosome 3B and *AX-110005500* at 568.20 Mb of chromosome 7D have not been reported before, so they might be presumed to be new stable genetic loci for grain hardness.

## Figures and Tables

**Table 1:** Descriptive statistics of three types of wheat in different environments in the association panel.

Type	Environment	Min	Max	Mean	SD	*CV*%
Hard wheat	E1	38.00	75.00	62.86	5.42	8.62
	E2	54.00	74.00	64.57	4.53	7.01
	E3	48.00	83.00	66.08	6.97	10.55
	E4	56.00	73.00	63.88	4.75	7.44
	BLUP	60.19	72.64	63.95	3.26	5.10
Mix wheat	E1	47.00	61.00	55.14	3.38	6.12
	E2	41.00	63.00	55.49	5.11	9.21
	E3	42.00	70.00	55.78	5.74	10.28
	E4	39.00	65.00	55.05	4.74	8.60
	BLUP	47.99	59.95	55.18	2.95	5.34
Soft wheat	E1	15.00	42.00	27.19	5.66	20.81
	E2	15.00	44.00	28.90	5.71	19.77
	E3	15.00	42.00	28.44	6.12	21.53
	E4	21.00	64.00	32.69	7.99	24.44
	BLUP	19.44	40.91	29.74	4.64	15.59

**Table 2:** Analysis of variance of HI trait in 150 wheat accessions.

Type	Genotype	Environment	Genotype × Environment	*H* ^2^
Hardness wheat	1.68 *	3.62	0.98	0.63
Mix wheat	1.77 *	0.02	0.70	0.71
Soft wheat	3.02 ***	13.11 ***	0.853	0.81

*: Significant at *p* < 0.05; ***: Significant at *p* < 0.001, The same below.

**Table 3:** Stable locus information with a significant correlation of HI.

Classification Standard	SNP	Chr.	Position (Mb)	*p* Value	*R*^2^ (%)	Environment
Hardness Index	*AX-111028882*	1A	27.39–31.94	1.10 × 10^−4^–9.74 × 10^−4^	7.63–10.66	E1, E2, E3, E4, BLUP
	*AX-110005500*	7D	568.20	1.17 × 10^−4^–8.84 × 10^−4^	7.52–10.57	E1, E2, E3, E4, BLUP
Assignment	*AX-108789085*	1A	24.86–32.43	9.75 × 10^−5^–9.44 × 10^−4^	7.77–10.86	E1, E2, E3, BLUP
	*AX-108899745*	1A	42.55–49.23	9.35 × 10^−5^–9.31 × 10^−4^	7.72–11.01	E1, E2, E3, BLUP
	*AX-109815102*	3B	606.68	1.24 × 10^−4^–3.77 × 10^−4^	9.00–10.61	E1, E2, BLUP
	*AX-111673206*	4B	656.90–661.26	3.60 × 10^−4^–9.83 × 10^−4^	7.63–9.00	E1, E2, E4
	*AX-110005500*	7D	568.20	8.12 × 10^−5^–8.55 × 10^−4^	7.91–11.12	E1, E2, E3, BLUP

**Table 4:** Stable candidate gene function prediction.

SNP	Chr.	Position (Mb)	Gene	Gene Annotation or Coding Protein	Previously Reported
*AX-108789085*	1A	24.86–32.43	*TraesCS1A01G045700*	Glutathione S-transferase	*2013_Mergoum_3* [13]
*AX-108899745*	1A	42.55–49.23	*TraesCS1A01G062500*	Protease inhibitor/seed storage/lipid transfer protein family protein	*qHA1A.1* [34]
*AX-109815102*	3B	606.68	*TraesCS3B01G386000*	O-Glycosyl hydrolases family 17 protein	-
*AX-111673206*	4B	656.90–661.26	*TraesCS4B01G372800*	F-box family protein	*qHA4B.3* [27]
*AX-110005500*	7D	568.20	*TraesCS7D01G448100*	Protease inhibitor/seed storage/lipid transfer family protein	-

## Data Availability

All data generated or analysed during this study are included in this pubulished (and its additional files). There is no privacy or ethics involved in the research data. We are willing to share data.

## Supplementary Materials

The following supporting information can be downloaded at: https://www.mdpi.com/article/10.3390/genes14030672/s1, Table S1: Name, type, source and hardness phenotype values of the test material in different environments. Table S2: Statistics of average temperature, accumulated temperature, average relative humidity, monthly precipitation, monthly Sunshine duration, and monthly days of rainfallof four cultivation environments.
