# Supplementary material for: Genome-Wide Association Analysis of Grain Hardness in Common Wheat
Source: Genes (Basel). 2023 Mar 8;14(3):672. doi: 10.3390/genes14030672 (PMC10047947; doi:10.3390/genes14030672)
Supplement: Supplementary file 1 [file genes-14-00672-s001.zip › genes-2160479-supplementary.pdf]

**Table S1 Name, type, source and hardness phenotype values of the test material in different environments.**

| Code | Variety (line) | Type | E1-Mean | E2-Mean | E3-Mean | E4-Mean | BLUP-Mean | Origin |
|---|---|---|---|---|---|---|---|---|
| 1 | Jimai 22 | hard wheat | 66 | 58 | 62 | 65 | 62.39 | Shandong, China |
| 2 | Anke 1302 | hard wheat | 68 | 69 | 38 | 67 | 60.19 | Anhui, China |
| 3 | Anke 157 | hard wheat | 65 | 69 | 75 | 73 | 69.96 | Anhui, China |
| 4 | Annong 0711 | hard wheat | 67 | 66 | 68 | 71 | 67.52 | Anhui, China |
| 5 | Bainong 207 | mix wheat | 53 | 53 | 55 | 50 | 52.63 | Henan, China |
| 6 | Fanmai 5 hao | soft wheat | 28 | 29 | 31 | 31 | 30.18 | *Henan, China* |
| 7 | Hengjinmai 8 | mix wheat | 53 | 65 | 56 | 61 | 58.49 | Anhui, China |
| 8 | Huacheng 1688 | soft wheat | 26 | 57 | 26 | 28 | 34.57 | Anhui, China |
| 9 | Huacheng 2019 | mix wheat | 56 | 55 | 58 | 53 | 55.31 | Anhui, China |
| 10 | Huacheng 3366 | mix wheat | 57 | 58 | 51 | 56 | 55.31 | Anhui, China |
| 11 | Huacheng 859 | mix wheat | 52 | 60 | 61 | 61 | 58.24 | Anhui, China |
| 12 | Huacheng 863 | soft wheat | 21 | 26 | 26 | 24 | 24.81 | Anhui, China |
| 13 | Huaimai 22 | soft wheat | 25 | 26 | 19 | 31 | 25.78 | Jiangsu, China |
| 14 | Huaimai 29 | mix wheat | 60 | 46 | 54 | 56 | 53.85 | Jiangsu, China |

| | | | | | | | | |
|---|---|---|---|---|---|---|---|---|
| 15 | Huaimai 30 | mix wheat | 57 | 56 | 52 | 51 | 53.85 | Jiangsu, China |
| 16 | Huaimai 33 | hard wheat | 69 | 58 | 61 | 62 | 62.15 | Jiangsu, China |
| 17 | Huaimai 35 | soft wheat | 32 | 33 | 28 | 31 | 31.40 | Jiangsu, China |
| 18 | Huaimai 40 | hard wheat | 64 | 63 | 69 | 67 | 65.32 | Jiangsu, China |
| 19 | Huaimai 44 | mix wheat | 59 | 56 | 59 | 59 | 58.00 | Jiangsu, China |
| 20 | Huiyan 66 | hard wheat | 58 | 59 | 60 | 66 | 60.44 | Anhui, China |
| 21 | Huiyan 912 | soft wheat | 33 | 31 | 22 | 28 | 28.95 | Anhui, China |
| 22 | Jimai 44 | mix wheat | 64 | 58 | 54 | 62 | 59.22 | Shandong, China |
| 23 | Lemai 2 | mix wheat | 54 | 50 | 47 | 41 | 47.99 | Anhui, China |
| 24 | Lianmai 2 | hard wheat | 69 | 56 | 57 | 61 | 60.44 | Jiangsu, China |
| 25 | Longke 0901 | soft wheat | 42 | 37 | 31 | 36 | 36.76 | Anhui, China |
| 26 | Longke 1109 | soft wheat | 25 | 34 | 25 | 24 | 27.49 | Anhui, China |
| 27 | Longke 1221 | hard wheat | 69 | 58 | 56 | 63 | 61.17 | Anhui, China |
| 28 | Luyuan 502 | hard wheat | 66 | 62 | 60 | 67 | 63.37 | Shandong, China |
| 29 | Mingmai 1hao | soft wheat | 34 | 30 | 29 | 31 | 31.40 | Jiangsu, China |
| 30 | Nongmai 152 | hard wheat | 65 | 59 | 63 | 60 | 61.42 | Jiangsu, China |

| | | | | | | | | |
|---|---|---|---|---|---|---|---|---|
| 31 | Qingnong 3 | hard wheat | 67 | 59 | 64 | 66 | 63.61 | Shandong, China |
| 32 | Quanmai 725 | soft wheat | 27 | 23 | 20 | 15 | 21.88 | Anhui, China |
| 33 | Ruihuamai 516 | soft wheat | 39 | 31 | 29 | 29 | 32.37 | Jiangsu, China |
| 34 | Ruihuamai 520 | mix wheat | 69 | 54 | 56 | 62 | 59.95 | Jiangsu, China |
| 35 | Ruihuamai 618 | hard wheat | 74 | 56 | 59 | 63 | 62.64 | Jiangsu, China |
| 36 | Shannong 17 | hard wheat | 83 | 65 | 63 | 65 | 68.49 | Shandong, China |
| 37 | Shannong 20 | hard wheat | 72 | 65 | 60 | 74 | 67.27 | Shandong, China |
| 38 | Sui 1216 | soft wheat | 30 | 33 | 23 | 34 | 30.42 | Shandong, China |
| 39 | Tainong 19 | hard wheat | 74 | 69 | 64 | 74 | 69.71 | Shandong, China |
| 40 | Tianyikemai 5 | soft wheat | 28 | 32 | 23 | 32 | 29.20 | Anhui, China |
| 41 | Wankenmai 0622 | mix wheat | 60 | 56 | 52 | 55 | 55.56 | Anhui, China |
| 42 | Wankenmai 1 hao | soft wheat | 31 | 25 | 18 | 20 | 24.07 | Anhui, China |
| 43 | Wankenmai 869 | mix wheat | 70 | 47 | 56 | 61 | 58.24 | Anhui, China |
| 44 | Wanmai 52 | soft wheat | 26 | 29 | 20 | 26 | 25.78 | Anhui, China |
| 45 | Weilai 0818 | soft wheat | 28 | 34 | 20 | 25 | 27.25 | Anhui, China |
| 46 | Guomai 99 | soft wheat | 25 | 64 | 19 | 28 | 34.32 | Anhui, China |

| | | | | | | | | |
|---|---|---|---|---|---|---|---|---|
| 47 | Guomai 9 | hard wheat | 65 | 58 | 59 | 65 | 61.42 | Anhui, China |
| 48 | Xinmai 26 | hard wheat | 62 | 60 | 63 | 63 | 61.66 | Henan, China |
| 49 | Su 553 | hard wheat | 57 | 62 | 60 | 69 | 61.66 | Anhui, China |
| 50 | Xunong 029 | hard wheat | 71 | 56 | 60 | 69 | 63.61 | Jiangsu, China |
| 51 | Yannong 19 | hard wheat | 77 | 70 | 64 | 71 | 69.96 | Shandong, China |
| 52 | Yannong 5158 | soft wheat | 38 | 34 | 32 | 38 | 35.79 | Shandong, China |
| 53 | Yannong 999 | soft wheat | 28 | 34 | 27 | 30 | 30.18 | Shandong, China |
| 54 | Zheng 7698 | hard wheat | 71 | 68 | 68 | 66 | 67.76 | Henan, China |
| 55 | Zhongmai 578 | mix wheat | 56 | 62 | 57 | 60 | 58.49 | Henan, China |
| 56 | Zhongmai 895 | mix wheat | 52 | 52 | 52 | 55 | 52.63 | Henan, China |
| 57 | Huaimai 45 | mix wheat | 58 | 55 | 55 | 58 | 56.29 | Jiangsu, China |
| 58 | Zhoumai 27 | mix wheat | 61 | 39 | 61 | 60 | 55.07 | Henan, China |
| 59 | Zhoumai 36 | mix wheat | 52 | 52 | 54 | 45 | 50.68 | Henan, China |
| 60 | Zimai 19 | soft wheat | 22 | 32 | 22 | 28 | 26.51 | Anhui, China |
| 61 | Guohong 6 | soft wheat | 22 | 31 | 28 | 24 | 26.76 | Anhui, China |
| 62 | Haomai 1 | soft wheat | 41 | 35 | 31 | 33 | 35.30 | Fujian, China |

| | | | | | | | | |
|---|---|---|---|---|---|---|---|---|
| 63 | Huamai 1028 | soft wheat | 22 | 29 | 20 | 21 | 23.59 | Jiangsu, China |
| 64 | Huamai 7 | mix wheat | 45 | 60 | 53 | 51 | 52.14 | Jiangsu, China |
| 65 | Longkenmai 1 | soft wheat | 17 | 31 | 15 | 26 | 22.85 | Anhui, China |
| 66 | Lunxuan 22 | hard wheat | 76 | 56 | 64 | 55 | 62.39 | Anhui, China |
| 67 | Lunxuan 27 | soft wheat | 29 | 23 | 19 | 21 | 23.59 | Anhui, China |
| 68 | Luomai 10 | mix wheat | 56 | 56 | 53 | 55 | 54.83 | Anhui, China |
| 69 | Mingmai 133 | hard wheat | 56 | 71 | 60 | 66 | 62.88 | Jiangsu, China |
| 70 | Ningmai 13 | hard wheat | 52 | 67 | 62 | 65 | 61.17 | Jiangsu, China |
| 71 | Ningmai 21 | soft wheat | 20 | 32 | 22 | 27 | 25.78 | Jiangsu, China |
| 72 | Ningmai 26 | hard wheat | 48 | 67 | 63 | 65 | 60.44 | Jiangsu, China |
| 73 | Nongmai 126 | soft wheat | 18 | 32 | 24 | 28 | 26.03 | Jiangsu, China |
| 74 | Nuo 1012 | hard wheat | 61 | 63 | 63 | 63 | 62.15 | Anhui, China |
| 75 | Nuo 1019 | soft wheat | 38 | 43 | 35 | 39 | 38.96 | Anhui, China |
| 76 | Sulong 128 | soft wheat | 22 | 37 | 32 | 35 | 31.88 | Zhejiang, China |
| 77 | Sumai 11 | soft wheat | 25 | 40 | 32 | 36 | 33.59 | Jiangsu, China |
| 78 | Sumai 10 | soft wheat | 23 | 37 | 35 | 33 | 32.37 | Jiangsu, China |

| | | | | | | | | | |
|---|---|---|---|---|---|---|---|---|---|
| 79 | Sumai 188 | soft wheat | 22 | 29 | 27 | 31 | 27.73 | Jiangsu, China |
| 80 | Wanmai 606 | soft wheat | 15 | 22 | 20 | 18 | 19.44 | Anhui, China |
| 81 | Wanximai 0638 | soft wheat | 30 | 31 | 29 | 26 | 29.44 | Anhui, China |
| 82 | Yangfumai 6 | soft wheat | 32 | 32 | 31 | 29 | 31.40 | Jiangsu, China |
| 83 | Yangfumai 7 | soft wheat | 25 | 29 | 25 | 27 | 27.00 | Jiangsu, China |
| 84 | Yangfumai 8 | soft wheat | 24 | 30 | 30 | 31 | 29.20 | Jiangsu, China |
| 85 | Yangmai 13 | soft wheat | 19 | 23 | 20 | 24 | 22.12 | Jiangsu, China |
| 86 | Yangmai 15 | soft wheat | 18 | 29 | 25 | 29 | 25.78 | Jiangsu, China |
| 87 | Yangmai 16 | hard wheat | 64 | 63 | 61 | 58 | 61.17 | Jiangsu, China |
| 88 | Yangmai 20 | soft wheat | 31 | 29 | 29 | 27 | 29.44 | Jiangsu, China |
| 89 | Yangmai 22 | soft wheat | 34 | 35 | 32 | 39 | 35.30 | Jiangsu, China |
| 90 | Yangmai 24 | soft wheat | 26 | 28 | 26 | 33 | 28.71 | Jiangsu, China |
| 91 | Yangmai 25 | soft wheat | 34 | 63 | 33 | 33 | 40.91 | Jiangsu, China |
| 92 | Yangmai 27 | soft wheat | 30 | 31 | 32 | 33 | 31.88 | Jiangsu, China |
| 93 | Yangmai 28 | hard wheat | 66 | 61 | 62 | 60 | 61.90 | Jiangsu, China |
| 94 | Yimai 9 | mix wheat | 59 | 52 | 51 | 55 | 54.09 | Henan, China |

| | | | | | | | | |
|---|---|---|---|---|---|---|---|---|
| 95 | Zhenmai 11 | soft wheat | 29 | 26 | 24 | 26 | 26.76 | Jiangsu, China |
| 96 | Zhenmai 12 | hard wheat | 80 | 73 | 71 | 69 | 72.64 | Jiangsu, China |
| 97 | Zhenmai 168 | hard wheat | 77 | 70 | 72 | 64 | 70.20 | Jiangsu, China |
| 98 | Zhenmai 9 | hard wheat | 72 | 66 | 63 | 63 | 65.56 | Jiangsu, China |
| 99 | 18A101 | mix wheat | 52 | 57 | 52 | 49 | 52.38 | Anhui, China |
| 100 | 18A104 | soft wheat | 31 | 38 | 34 | 31 | 33.84 | Anhui, China |
| 101 | 18A105 | hard wheat | 62 | 63 | 66 | 62 | 62.88 | Anhui, China |
| 102 | 18A107 | hard wheat | 62 | 62 | 65 | 67 | 63.61 | Anhui, China |
| 103 | 18A108 | soft wheat | 28 | 38 | 26 | 34 | 31.88 | Anhui, China |
| 104 | 18A111 | mix wheat | 48 | 52 | 55 | 56 | 52.63 | Anhui, China |
| 105 | 18A126 | hard wheat | 61 | 62 | 63 | 61 | 61.42 | Anhui, China |
| 106 | 18A130 | hard wheat | 63 | 66 | 65 | 60 | 63.12 | Anhui, China |
| 107 | 18B130 | hard wheat | 62 | 64 | 61 | 62 | 61.90 | Anhui, China |
| 108 | 18B128 | hard wheat | 62 | 63 | 58 | 59 | 60.19 | Anhui, China |
| 109 | 18B132 | hard wheat | 72 | 70 | 68 | 61 | 67.27 | Anhui, China |
| 110 | 18B141 | mix wheat | 62 | 58 | 55 | 57 | 57.75 | Anhui, China |

| | | | | | | | | |
|---|---|---|---|---|---|---|---|---|
| 111 | 18B151 | hard wheat | 60 | 61 | 63 | 65 | 61.90 | Anhui, China |
| 112 | 18B177 | mix wheat | 52 | 56 | 52 | 48 | 51.90 | Anhui, China |
| 113 | 18B186 | mix wheat | 54 | 55 | 58 | 59 | 56.29 | Anhui, China |
| 114 | 18B187 | mix wheat | 53 | 58 | 55 | 59 | 56.05 | Anhui, China |
| 115 | 18B189 | hard wheat | 58 | 68 | 62 | 63 | 62.39 | Anhui, China |
| 116 | 18B190 | hard wheat | 61 | 62 | 63 | 65 | 62.39 | Anhui, China |
| 117 | 18B191 | soft wheat | 27 | 31 | 33 | 21 | 28.47 | Anhui, China |
| 118 | 18B194 | soft wheat | 25 | 26 | 22 | 23 | 24.56 | Anhui, China |
| 119 | 18B210 | soft wheat | 37 | 33 | 35 | 31 | 34.32 | Anhui, China |
| 120 | 18B223 | mix wheat | 59 | 55 | 59 | 54 | 56.53 | Anhui, China |
| 121 | 18B233 | mix wheat | 56 | 54 | 61 | 63 | 58.24 | Anhui, China |
| 122 | 18B248 | mix wheat | 56 | 56 | 59 | 61 | 57.75 | Anhui, China |
| 123 | 18B257 | soft wheat | 27 | 29 | 31 | 22 | 27.73 | Anhui, China |
| 124 | 18B259 | mix wheat | 51 | 55 | 53 | 51 | 52.38 | Anhui, China |
| 125 | 18B369 | mix wheat | 56 | 62 | 58 | 58 | 58.24 | Anhui, China |
| 126 | 18B375 | soft wheat | 35 | 32 | 28 | 34 | 32.62 | Anhui, China |

| | | | | | | | | |
|---|---|---|---|---|---|---|---|---|
| 127 | CP03-28-1 | hard wheat | 68 | 69 | 71 | 73 | 69.71 | Beijing, China |
| 128 | Chuan 42 | mix wheat | 53 | 51 | 55 | 54 | 53.12 | Sichuan, China |
| 129 | Yangmai 158 | mix wheat | 48 | 53 | 57 | 56 | 53.36 | Jiangsu, China |
| 130 | Anke 1604 | mix wheat | 62 | 59 | 61 | 58 | 59.71 | Anhui, China |
| 131 | CP01-27-3 | hard wheat | 61 | 59 | 71 | 72 | 65.32 | Beijing, China |
| 132 | Yangnuomai 1 | soft wheat | 36 | 34 | 33 | 35 | 34.81 | Jiangsu, China |
| 133 | 950218-54 | soft wheat | 33 | 36 | 36 | 31 | 34.32 | Anhui, China |
| 134 | Bainong 64 | soft wheat | 28 | 29 | 23 | 18 | 25.05 | Henan, China |
| 135 | External citation material | mix wheat | 42 | 58 | 52 | 48 | 49.94 | Israel |
| 136 | Huaanmai 825 | soft wheat | 33 | 35 | 35 | 32 | 34.08 | Anhui, China |
| 137 | Chuanmai 42 | soft wheat | 33 | 36 | 31 | 29 | 32.62 | Sichuan, China |
| 138 | Xinyuan 958 | hard wheat | 56 | 61 | 63 | 64 | 60.68 | Henan, China |
| 139 | Zhongkemai 138 | hard wheat | 69 | 65 | 66 | 61 | 64.83 | Sichuan, China |
| 140 | Chuannong 16 | soft wheat | 25 | 21 | 33 | 35 | 28.95 | Sichuan, China |
| 141 | Xikemai 8 | hard wheat | 63 | 67 | 58 | 54 | 60.19 | Sichuan, China |
| 142 | Mianmai 267 | soft wheat | 31 | 39 | 32 | 28 | 32.86 | Sichuan, China |

| | | | | | | | | |
|---|---|---|---|---|---|---|---|---|
| 143 | 21946 | hard wheat | 67 | 73 | 61 | 63 | 65.56 | Sichuan, China |
| 144 | Xinong 916 | hard wheat | 68 | 71 | 59 | 65 | 65.32 | Shaanxi, China |
| 145 | B12-6-1 | hard wheat | 69 | 62 | 61 | 58 | 62.15 | Sichuan, China |
| 146 | Mianmai 37 | soft wheat | 33 | 28 | 22 | 19 | 26.03 | Sichuan, China |
| 147 | Chuanmai 82 | soft wheat | 39 | 36 | 42 | 44 | 40.43 | Sichuan, China |
| 148 | F06-4198 | mix wheat | 57 | 56 | 51 | 55 | 54.58 | Sichuan, China |
| 149 | 2011 | hard wheat | 75 | 68 | 68 | 63 | 68.00 | Sichuan, China |
| 150 | 206A | soft wheat | 28 | 25 | 24 | 27 | 26.51 | Anhui, China |

**Table S2** Statistics of average temperature, accumulated temperature, average relative humidity, monthly precipitation, monthly Sunshine duration, and monthly days of rainfallof four cultivation environments

| Meteorological data | Location | October | November | December | January | February | March | April | May |
|---|---|---|---|---|---|---|---|---|---|
| Average temperature (℃) | E1 | 13.95 | 10.39 | 4.44 | -0.65 | 4.17 | 11.42 | 16.69 | 21.46 |
| | E2 | 15.11 | 9.86 | 3.42 | 1.81 | 2.79 | 11.44 | 15.52 | 21.23 |
| | E3 | 16.32 | 12.41 | 5.57 | 3.37 | 7.74 | 11.83 | 15.70 | 23.67 |
| | E4 | 15.51 | 11.49 | 3.25 | 3.90 | 9.28 | 11.06 | 15.26 | 21.73 |
| | Taihe two-year average | 14.53 | 10.13 | 3.93 | 0.58 | 3.48 | 11.43 | 16.11 | 21.35 |
| | Funan two-year average | 15.92 | 11.95 | 4.41 | 3.64 | 8.51 | 11.45 | 15.48 | 22.70 |
| Effective temperature（℃） | E1 | 237.20 | 311.80 | 138.70 | 36.50 | 126.00 | 353.90 | 500.60 | 665.30 |
| | E2 | 256.90 | 295.70 | 124.00 | 62.10 | 83.60 | 354.70 | 465.70 | 658.20 |
| | E3 | 277.40 | 372.30 | 174.20 | 104.40 | 224.40 | 366.60 | 470.90 | 733.70 |
| | E4 | 263.70 | 344.60 | 112.60 | 134.90 | 259.90 | 342.80 | 457.80 | 673.70 |
| | Taihe two-year average | 247.05 | 303.75 | 131.35 | 49.30 | 104.80 | 354.30 | 483.15 | 661.75 |
| | Funan two-year average | 270.55 | 358.45 | 143.40 | 119.65 | 242.15 | 354.70 | 464.35 | 703.70 |
| Average relative humidity（%） | E1 | 80.35 | 67.77 | 64.29 | 80.35 | 68.96 | 79.00 | 74.93 | 82.84 |

| | | | | | | | | | |
|---|---|---|---|---|---|---|---|---|---|
| | E2 | 69.76 | 84.63 | 79.23 | 75.35 | 82.43 | 65.81 | 78.00 | 68.77 |
| | E3 | 60.18 | 62.17 | 73.39 | 85.61 | 74.66 | 72.19 | 65.80 | 66.06 |
| | E4 | 72.65 | 73.50 | 71.06 | 64.10 | 73.89 | 79.39 | 77.93 | 73.48 |
| | Taihe two-year average | 75.06 | 76.20 | 71.76 | 77.85 | 75.70 | 72.41 | 76.47 | 75.81 |
| | Funan two-year average | 66.42 | 67.84 | 72.23 | 74.86 | 74.28 | 75.79 | 71.87 | 69.77 |
| Monthly precipitation (mm) | E1 | 15.80 | 5.70 | 3.00 | 49.50 | 18.20 | 34.30 | 44.80 | 163.90 |
| | E2 | 1.40 | 83.30 | 39.90 | 26.80 | 24.10 | 58.90 | 36.50 | 4.80 |
| | E3 | 0.50 | 21.90 | 10.10 | 119.60 | 56.00 | 107.90 | 13.00 | 8.30 |
| | E4 | 14.00 | 62.10 | 13.20 | 19.60 | 29.50 | 77.90 | 33.90 | 95.20 |
| | Taihe two-year average | 8.60 | 44.50 | 21.45 | 38.15 | 21.15 | 46.60 | 40.65 | 84.35 |
| | Funan two-year average | 7.25 | 42.00 | 11.65 | 69.60 | 42.75 | 92.90 | 23.45 | 51.75 |
| Monthly Sunshine duration (h) | E1 | 90.50 | 173.20 | 103.70 | 76.00 | 144.70 | 164.70 | 222.30 | 181.60 |
| | E2 | 112.00 | 118.60 | 81.40 | 73.90 | 54.80 | 199.20 | 162.50 | 203.70 |
| | E3 | 115.70 | 151.90 | 155.80 | 66.00 | 141.50 | 165.50 | 213.10 | 231.40 |
| | E4 | 81.00 | 149.70 | 156.30 | 154.60 | 171.90 | 139.20 | 158.80 | 200.50 |
| | Taihe two-year average | 101.25 | 145.90 | 92.55 | 74.95 | 99.75 | 181.95 | 192.40 | 192.65 |

| | | | | | | | | | |
|---|---|---|---|---|---|---|---|---|---|
| | Funan two-year average | 98.35 | 150.80 | 156.05 | 110.30 | 156.70 | 152.35 | 185.95 | 215.95 |
| Monthly days of rainfall（day） | E1 | 4.00 | 3.00 | 1.00 | 10.00 | 7.00 | 9.00 | 8.00 | 16.00 |
| | E2 | 2 | 8 | 10 | 6 | 13 | 3 | 8 | 4 |
| | E3 | 1 | 8 | 7 | 14 | 9 | 10 | 6 | 2 |
| Average temperature (℃) | E4 | 2 | 9 | 5 | 4 | 6 | 16 | 10 | 10 |
| | Taihe two-year average | 3 | 6 | 6 | 8 | 10 | 6 | 8 | 10 |
| | Funan two-year average | 2 | 9 | 6 | 9 | 8 | 13 | 8 | 6 |

**Note:** E1, E2, E3, E4 represent 2017-2018, 2018-2019 Taihe and 2019-2020, 2020-2021 Funan, respectively.According to the sowing time of each year, the average temperature, accumulated temperature, average relative humidity, monthly precipitation, monthly Sunshine duration, and monthly days of rainfallof four cultivation environments data of October are counted from 15th to 31st, and the data of November to May are counted in the whole month.Data from: Anhui Meteorogical Bureau.
